# Does the novel coronavirus use the ocular surface as an entrance into the body or as an infection site?

**DOI:** 10.5935/0004-2749.20220012

**Published:** 2022

**Authors:** Remzi Karadag, Alp Kayiran, Christopher J. Rapuano

**Affiliations:** 1 Veni Vidi Eye Center, Caddebostan, Kadikoy, Istanbul, Turkey; 2 Department of Ophthalmology, Yeditepe University School of Medicine, Istanbul, Turkey; 3 Cornea Service, Wills Eye Hospital, Sidney Kimmel Medical College at Thomas Jefferson University, Philadelphia, USA

**Keywords:** Coronavirus infections, Coronavirus, COVID-19, SARS-CoV-2, Conjunctivitis, Eye manifestations, Disease transmission, infectious, Infecções por coronavirus, Coronavirus, COVID-19, SARS-CoV-2, Conjuntivite, Manifestações oculares, Transmissão de doença infecciosa

## Abstract

This study attempts to review whether the coronavirus disease-2019 (COVID-19) is transmitted through the ocular surface and examine the symptoms and signs of ocular disease. Considering that COVID-19 is transmitted by airborne droplets and close contact with infected individuals, we will also review the conditions to which eye clinics and ophthalmologists should pay attention to prevent the transmission of the disease. Although some researchers have argued that COVID-19 transmission cannot occur through the ocular surface, most of them are of the opinion that the ocular surface is a potential pathway of transmission. Until date, ocular signs and symptoms have been rarely reported in the COVID-19 patients. However, there are case reports of conjunctivitis as the first, and rarely, the only clinical symptom of the disease. In addition, low coronavirus RNA positivity can be detected in the ocular surface samples. Further laboratory and clinical investigations are needed to ascertain whether the ocular surface is one of the potential transmission pathways through which severe acute respiratory syndrome-coronavirus 2 can gain entry into the human body.

## Abstract (pt)

Rever se o COVID- 19 é transmitido através da superfície ocular e seus sintomas e sinais na doença ocular. Dado que o COVID-19 é transmitido por gotículas de ar e contato próximo, também analisaremos, também, as condições às quais os oftalmologistas e as clínicas oftalmológicas devem prestar atenção a fim de evitar a transmissão da doença. Embora alguns autores tenham argumentado que a transmissão de COVID-19 não pode ocorrer através da superfície ocular, a maioria dos autores acredita que a superfície ocular é uma via potencial de transmissão. Até à data, foram notificados, muito raramente, sinais e sintomas oculares em doentes com COVID- 19. No entanto, há relatos de casos de conjuntivite como sendo, raramente, o primeiro e único sintoma clínico da doença. Além disso, a baixa positividade do RNA coronavírus pode ser detectada nas amostras da superfície ocular. São necessárias mais investigações clínicas laboratoriais sobre se a superfície ocular é uma das vias de transmissão através das quais o SARS-COV-2 penetra no corpo humano.

## INTRODUCTION

The coronavirus disease 2019 (COVID-19), which was first reported in the city of Wuhan in the Hubei Province of the People’s Republic of China in 2019, is caused by severe acute respiratory syndrome-coronavirus-2 (SARS-CoV-2)^(1-4)^. COVID-19 was declared a pandemic by the World Health Organization on March 11, 2020. As of June 2, 2020, there are 6,382,952 confirmed cases and 380,318 fatalities worldwide^(5)^.

Although COVID-19 mainly affects the respiratory system, it may also involve other organ systems such as the gastrointestinal, olfactory, neurological, renal, cardiovascular, and ocular systems^(1,2)^.

This article aims to review the methods of contamination as well as the clinical and laboratory ocular findings of COVID-19 and highlight the currently available eye protection methods.

### Severe acute respiratory syndrome-coronavirus 2

Coronaviruses (CoVs) are known to affect a wide range of birds and mammals, including pets such as cats (feline) and dogs (canine)^(6,7)^. CoVs are zoonotic pathogens capable of mutating, which facilitates their transmission from animals to humans^(8)^.

SARS-CoV-2, Middle East respiratory syndrome-coronavirus (MERS-CoV), and severe acute respiratory syndrome-coronavirus (SARS-CoV) belong to the family of beta Coronaviridae which are enveloped positive-stranded ribonucleic acid (RNA) viruses. There are four groups of CoVs, namely alpha, beta, gamma, and delta. While the alpha and beta coronaviruses primarily affect the respiratory, gastrointestinal, and central nervous systems of human and mammalian hosts, gamma and delta coronaviruses are mostly avian pathogens^(1-3)^. Coronaviruses are capable of interspecies transmission and cause diseases ranging from the common cold to the more severe MERS and SARS in humans.

## CONTAMINATIONS

### Frequent methods of contamination

The main routes of transmission for human coronaviruses are thought to be respiratory droplets generated by infected individuals and direct contact with virus-contaminated fomites. Recently, SARS-CoV and SARS-CoV-2 have also been detected in stool and urine samples, making the fecal-oral route a possible means of transmission for the viruses. Additionally, transconjunctival contamination has been hypothesized since the main contamination route of SARS-CoV-2 is trans mucosal^(1-4)^.

The virus can remain viable on diverse surfaces for up to 72 hours with different degrees of infectivity^(1)^. The median incubation period is 5.1 days and ranges from 1 to 14 days^(1-4)^.

The coronavirus binds to specific receptors on host cells through its S protein and enters them. Angiotensin-converting enzyme-2 (ACE-2) is a cellular receptor that is utilized by SARS-CoV-2 to enter the cell. The entry receptor of SARS-CoV, HCoV-NL63, and SARS-CoV-2 is ACE2, which is highly expressed in the human lung alveolar epithelial cells, enterocytes of the intestinal system, and the proximal tubular cells of the kidney^(9,10)^.

Viral entry is also associated with type II transmembrane serine protease (TMPRSS2) activity. Although it has been reported that ACE-2 is not present in the upper airway, ACE-2 and TMPRSS-2 have been detected in the epithelial cells of the upper and lower airways by immunohistochemical methods. Specifically, the gene expression patterns of these two receptors have de monstrated that alveolar type II epithelial cells are largely involved^(9,10)^. Kam et al.^(11)^ reported that entry into human B cell lines involves a FcγRII-dependent and ACE2-independent mechanism, showing that antibody-dependent enhancement (ADE) of viral entry is another potential target cell entryway of SARS-CoV. ADE occurs when a virus-antibody complex interacts with Fc Receptor or complement to begin viral uptake or alternatively when antibodies stimulate structural changes in envelope glycoproteins that are needed for virus-cell membrane fusion^(11)^. In another study, Yang et al.^(12)^ showed that a civet cat type of SARS-CoV demonstrated enhanced entry into a human renal epithelial cell-line in the presence of human monoclonal antibodies against a human type of SARS-CoV spike.

### Transmission via the ocular surface

Since the main transmission route of SARS-CoV-2 is trans mucosal, ocular surface transmission is one of the contamination pathways that has been speculated by many researchers. Anatomically, the ocular surface carries a risk of exposure to infectious droplets and fomites during close contact with patients or contaminated hands. The results from the limited number of studies on this subject remain controversial^(9,13-17)^.

Conjunctivitis and other ocular complications have not been reported in patients with SARS-CoV and MERS-CoV disease, while few cases with SARS-CoV-2 infection and only four cases with HCo-NL63 infection have been reported to be associated with conjunctivitis^(9,13-17)^. Therefore, ocular manifestations are rarely seen in human CoV infection^(8,10,13-16)^.

Recently, the presence of human CoV RNA in ocular surface samples was tested by reverse transcription-polymerase chain reaction (RT-PCR) in SARS and COVID-19 patients; however, the positivity rate was exceedingly low^(4,16-20)^. Deng et al. reported that no SARS-CoV-2 could be detected by RT-PCR in 114 ocular surface samples obtained from patients with COVID-19 pneumonia^(21)^. Additionally, Lange et al. evaluated the mRNA expression pattern of the SARS-CoV-2 receptor ACE-2 and its cofactors in 38 human conjunctival samples. The findings indicated that the SARS-CoV-2 receptor ACE-2 and other auxiliary molecules are not substantially expressed in conjunctival samples at the mRNA or protein levels^(22)^. Because of the rarity of the ocular manifestations and the extremely low positivity rate of human CoV RNA test by RT-PCR in ocular surface samples, ocular surface transmission is not considered by some authors to be a prime contamination route of human CoV infection^(4,13-17)^.

The human eye has its own intraocular renin-angiotensin system, which exists not only on the ocular surface but also inside the eye (trabecular meshwork, aqueous humor, iris, ciliary body, and retina). Similar to other cells in the body, ACE2 and TMPRSS2 are currently believed to be important factors in ocular surface cell entry^(23-26)^. Previous studies elicited significant gene expression of the ACE-2 receptor in the ocular surface together with TMPRSS2 protein. The co-expression of ACE-2 and TMPRSS2 in the corneal limbal cells verifies the high affinity of this tissue for SARS-CoV-2 and its existence in tears^(23-26)^. Although the expression of ACE-2 was detected in ocular surface epithelial cells, its expression in the corneal and conjunctival epithelial cells was found to be lower than that in the lung and kidney cells^(10,25,26)^.

The nasolacrimal system provides an anatomic connection between the ocular surface and the upper respiratory tract. When a drop is instilled into the eye, even though some of it is absorbed by the cornea and the conjunctiva, most of it is drained into the nasal cavity through the nasolacrimal canal and is subsequently transferred to the upper respiratory or the gastrointestinal tract^(27)^. The presence of SARS-CoV-2 in tears has also been reported. Hence, the passage of the virus from the eye to the nasopharynx via a patent nasolacrimal canal is possible regardless of the presence of ACE2 receptors in the cornea and conjunctiva^(28)^.

Loon et al. detected SARS-CoV in the tear samples of 36 consecutive suspects during the SARS epidemic in 2004^(14)^. SARS-CoV was present in the tears only in three of the eight confirmed SARS cases sampled in the early phase of their illness. In other cases, who were in the later phase of the illness, the tear samples were SARS-CoV negative^(18)^. Conjunctival swab and sputum samples of 30 COVID-19 patients with conjunctivitis were analyzed by PCR for SARS-CoV-2 in a prospective study by Xia et al.^(17)^. Each patient was sampled twice, with an interval of 2-3 days. Only two conjunctival and tear samples in the eye with conjunctivitis were positive, while 55 of the 60 sputum samples showed positive PCR results for SARS-CoV-2^(17)^. Previous studies revealed the presence of SARS-CoV in patients’ tears and MERS-CoV RNA in camels’ conjunctival swabs in the early stage of the disease using PCR. Both of these viruses are members of the same Coronaviridae family to which SARS-CoV-2 belongs^(18,29)^.

Chen et al.^(30)^ detected SARS-CoV-2 in the conjunctival swabs of a patient with confirmed COVID-19 and bilateral conjunctivitis 13 days after the onset of the illness. Viral RNA was found in their patient’s conjunctiva for at least 5 days. However, the authors stated that the viral RNA levels in conjunctival specimens were significantly lower than those in airway samples^(30)^. Wu et al.^(31)^ retrospectively evaluated 38 patients with either confirmed COVID-19 or highly suspected to have the disease. They reported that the RT-PCR results were positive for SARS-CoV-2 in 28 nasopharyngeal swabs but only in 2 conjunctival swabs although 12 of their patients had ocular manifestations. The authors stated that the low positivity rates of the conjunctival swab results might have been due to the fact that only one sample was taken from each patient’s eye^(31)^.

Deng et al.^(32)^ showed that SARS-CoV-2 infection could be induced by ocular surface inoculation in an experimental animal model using macaques. Although the researchers detected the virus in conjunctival swabs only on the first day after inoculation, they continued to detect it in nasal and throat swabs 1-7 days after the inoculation. Their findings demonstrated that the viral load in the airway mucosa was much higher than that in the ocular surface. They euthanized and necropsied one of the conjunctival inoculated-animals and found that the virus had spread to the nasolacrimal system and ocular tissue, nasal cavity, pharynx, trachea, tissues in the oral cavity, tissues in the lower-left lobe of the lung, inguinal and perirectal lymph node, stomach, duodenum, cecum, and ileum. They also found a specific IgG antibody, indicating that the animal was infected with SARS-CoV-2 via the ocular surface route^(32)^.

The remarkably low positivity rate of SARS-CoV-2 in the tears and conjunctival secretions of COVID-19 patients when tested by RT-PCR may be explained as follows: First, the sensitivity of the currently used RT-PCR test still needs to be improved because previous studies have reported that the sensitivity of the test is only 50%-60%^(19,27)^. Second, the time of ocular surface sample collection is likely to be in the later phase of the infection unless the patient exhibited ocular symptoms at the onset of infection. Previous studies demonstrated that samples with positive PCR results were mostly obtained within 7 days of the onset of infection. Since the ocular findings are infrequent in patients with SARS-CoV-2 RNA, it is highly likely that samples were either not taken at all or not taken at the appropriate time. Third, in current clinical practice, although some suspected COVID-19 cases often undergo 2-3 repeated tests of nasopharyngeal swabs before obtaining positive results^(14)^, the same number of repeated tests are not usually performed for ocular surface samples. Additionally, because the respiratory symptoms of COVID-19 infection are often severe and even fatal, mild eye symptoms are not prioritized, and therefore, ocular surface swabs may not be tested. Fourth, SARS-CoV-2 may originate from fomites and be transmitted to the ocular surface via splashed droplets or via direct contact with contaminated hands. The majority of these viruses are transmitted through the lacrimal canal to the nasopharynx owing to the washing effect of the tears^(1,32)^. Therefore, in individuals with ocular surface contact, the probability of a positive test may increase when tears and conjunctival samples are obtained at the earliest after the contact occurred^(33)^. Fifth, antimicrobial agents such as lactoferrin and secretory IgA, in addition to the washing effect of tears, reduce the viral load in the ocular surface^(27,34)^. Sixth, some swabs used in specimen sampling (calcium alginate swabs or swabs with wooden shafts) may contain substances that inactivate viruses and inhibit PCR testing. Moreover, topical anesthetics, which may affect the survival of the viruses, are not recommended while collecting ocular surface samples^(35)^. Lastly, insufficient tear collection during sampling may have some effect on the positivity rate of the PCR test^(1,34)^. In the light of previous publications, most authors believe that the ocular surface may be a potential but unlikely route of transmission for the coronavirus^(18,29-32)^.

## CLINICAL MANIFESTATIONS

The systemic clinical manifestations of COVID-19 include fever, chills, fatigue, muscle pain, headache, sore throat, loss of taste or smell, and respiratory distress. The clinical spectrum of the disease varies from asymptomatic and mild forms to respiratory failure that requires intensive care unit support, which may at times progress to sepsis, septic shock, and multiple organ dysfunction syndromes^(36)^. Up to 50% of the positive cases may be asymptomatic; thus, approximately 80% of the patients are either asymptomatic or show only mild symptoms^(36,37)^. In some cases, bilateral irregular shadows or frosted glass opacities have been demonstrated on the chest computed tomographic scans^(36)^.

The most common ocular manifestation of SARS-CoV-2 is mild conjunctivitis that presents with a follicular reaction, hyperemia, chemosis, watery discharge, and mild eyelid edema^(1)^. Although the prevalence of ocular manifestations among the COVID-19 patients has been reported to be <5% in many studies^(1,14,28)^, it has been demonstrated to be as high as 31.6% in one study^(31)^. The reason for the low rate of conjunctivitis reported by most studies may be the fact that mild conjunctivitis does not receive sufficient attention and is not well documented in patients presenting with a complex and life-threatening clinical scenario. Moreover, patients with conjunctivitis symptoms that are associated with mild systemic symptoms often quarantine themselves and may never undergo a medical evaluation. Interestingly, only 5% of the conjunctival swabs were positive for SARS-CoV-2 in a case series in which 31.6% of the patients had conjunctivitis^(31)^.

Wu et al.^(31)^ retrospectively evaluated the results of 38 patients confirmed or highly suspected to have COVID-19. Among them, 12 had various ocular manifestations, including epiphora, conjunctival congestion, or chemosis. Such ocular findings were generally noted in patients with more serious systemic manifestations^(31)^.

Chen et al.^(30)^ reported the case of a patient with confirmed COVID-19 and bilateral conjunctivitis. They found that the ocular findings occurred 13 days after the onset of systemic illness^(30)^. A case of pseudomembranous and hemorrhagic conjunctivitis was documented^(38)^. Salducci et al.^(39)^ reported a case of a person who had traveled from Italy to Japan with COVID-19 and developed severe viral conjunctivitis characterized by redness, irritation, and swelling in both eyes along with serous secretions, conjunctival chemosis, and pseudomembranes^(39)^.

As of now, there are detailed reports of five COVID-19 patients who demonstrated conjunctivitis as the initial finding of a systemic COVID-19 infection^(13,20,31,39,40)^. Two of them were health workers, i.e., a pulmonologist^(20)^ and an anesthesiologist^(13)^. The former, who had visited Wuhan in January 2020 reported that he was infected with SARS-CoV-2 despite being fully gowned with a protective suit and an N95 mask. Conjunctivitis was his first clinical manifestation, followed by catarrhal symptoms and fever a few hours later^(20)^. Shortly after this information was available, health care workers in China were encouraged to use eye protection during close contact with confirmed or suspected COVID-19 patients. The anesthesiologist who had conjunctivitis as the initial COVID-19 symptom stated that she had worn a surgical mask, cap, and gloves during patient care. Her ocular manifestations developed after she had performed tracheal intubation for a patient with COVID-19. She later developed fever and cough^(13)^. The other three patients with the initial symptom of conjunctivitis were non-healthcare professionals^(31,39,40)^. Even though all five patients had obvious ocular manifestations, the SARS-CoV-2 test was positive in ocular surface samples in only two of them^(13,20,31,39,40)^. Apart from these five patients, Stalinci and Battagliola reported five confirmed COVID-19 cases in whom conjunctivitis was the sole finding and no other systemic symptoms were present^(41)^.

Marinho et al. reported retinal OCT findings in confirmed COVID-19 patients who did not exhibit any ocular symptoms. All 12 patients in this series showed hyper-reflective lesions at the level of ganglion cell and inner plexiform layers prominently at the papillomacular bundles in both eyes, representing an inflammatory response to the virus^(42)^.

Given that conjunctivitis may be the first symptom of COVID-19, the source of the disease may well be the ocular surface^(13,20,31,39,40)^. However, most authors believe that the ocular surface is less likely to be the source of tissue invasion and ocular infection. Further studies are needed to resolve this issue.

## PROTECTION

As is evident from the review, there is no consensus among the authors regarding COVID-19 transmission via the ocular surface, and the available data are mostly based on anecdotal reports. However, since COVID-19 is a life-threatening disease, it is best to assume that it is transmissible through the ocular surface until additional evidence is available. Therefore, all ophthalmology departments and offices should take special precautions during this pandemic to protect their doctors, staff members, and patients. It is important to note that an ophthalmologist contracted the disease after contact with a glaucoma patient with subclinical COVID-19 and later lost his life^(43)^.

While most ocular disorders are not life-threatening, sight-threatening conditions warrant attention. Cases of ocular trauma, retinal detachment, acute angle-closure glaucoma, severe infections such as keratitis, orbital cellulitis, or uveitis exacerbations, and acute posterior vitreous detachment should be evaluated without any delay. If patients need emergency surgery, it should be done under local anesthesia if possible, and a COVID-19 test should be performed if the patient has fever or other suspicious symptoms. However, follow up examinations of slow-progressing disorders such as cataract, chronic open-angle glaucoma, or ptosis and procedures such as contact lens refitting may be put off for a reasonable time^(43)^.

The decision regarding intravitreal injections should be considered on a case-by-case basis. While temporarily postponing injections for diabetic macular edema and retinal branch vein occlusion might be acceptable, treatment plans for wet-type age-related macular degeneration and central retinal vein occlusion should not be altered^(44)^.

Care for posterior uveitis patients is of special significance since there is a potential threat of vision loss if the treatment is delayed. For newly diagnosed cases and acute exacerbations of chronic diseases, the patients may be treated with periocular steroid injections to delay systemic steroid therapy as the latter could potentially increase the morbidity and mortality risk during a pandemic. For patients in whom steroid treatment alone may suffice, such as those with Behçet’s disease necessitating the use of antimetabolites, calcineurin inhibitors, or biologic agents, dose lowering and periocular steroid injections may be considered^(45)^.

The number of appointments per day should be reduced, thereby allowing an adequate interval between the patients. Those with non-urgent complaints who do not have severe pain, vision loss, or photophobia may be assessed through teleconsultation. Timely telephonic or video consultations may avoid unnecessary visits by proper triage^(46)^.

All patients examined in an office should be questioned about possible COVID-19 symptoms such as fever, cough, shortness of breath, or loss of smell and taste along with possible contact with a COVID-19 patient. Temperature measurements are recommended before entering the examination room^(43,46,47)^.

The patients confirmed or suspected to have COVID-19 should be examined in a separate room with a minimum number of people in it. The patients must wear a mask and disinfect their hands. The physicians should be in full personal protective equipment, including gown, FFP2 or FFP3 mask, goggles, and gloves. Handwashing with soap and water for at least 20 seconds is crucial before and after each examination. The room should be properly ventilated after the examination, and the contact surfaces should be disinfected with 70% ethyl alcohol or the equivalent^(43)^.

The proximity between the patient and the physician during slit-lamp examination makes ophthalmologists particularly vulnerable to SARS-CoV-2 contamination. Transparent shields attached to the slit-lamp microscope, in addition to the standard protective equipment, may be helpful in avoiding the spread of aerosols. These shields should be disinfected with 70% ethyl alcohol or the equivalent after each examination^(47)^.

Reusable examination tools such as tonometer, A- and B-scan probes, and contact examination lenses should be properly disinfected. Automatic phoropters for refraction and indirect helmet ophthalmoscopes for retinal examination are preferable as they enable a distant examination. The top portion of the patient’s mask may be fixated with a skin plaster to avoid fogging of the oculars of the phoropter. Non-contact tonometer should be located in a separate room to avoid aerosol formation with the air puff^(44)^. Additionally, particular attention should be paid to washing hands frequently with soap and water or the equivalent^(43-47)^.

Although some authors have argued that COVID-19 transmission cannot occur through the ocular surface, the general opinion of most authors is that the ocular surface is a potential pathway of transmission. Since COVID-19 is a life-threatening disease, it is safe to assume that it may be transmitted through the ocular surface until sufficient laboratory and clinical data are available to prove otherwise. To date, ocular signs and symptoms have been rarely reported in COVID-19 patients. However, there are case reports of conjunctivitis as the first, and occasionally, the only clinical manifestation of the disease. Since COVID-19 is transmitted through droplets and by close contact with infected individuals and because ophthalmologists are in close proximity to their patients during the examination, they are at an increased risk for this infection. Therefore, patients, technicians, and physicians should use personal protective equipment during the process. Protective glasses, face mask/shield, and shield attached to a biomicroscope should be used to prevent conjunctival transmission. Moreover, the devices and tools used during the examination must be cleaned upon contact with a suspect. Further laboratory and clinical investigations are needed to ascertain whether the ocular surface is one of the transmission pathways through which SARS-CoV-2 enters the human body.
